# Lactic acid promotes non-small cell lung cancer progression by down-regulating hexokinase 1 via histone lactylation: A Mendelian randomization study

**DOI:** 10.1097/MD.0000000000045065

**Published:** 2025-10-03

**Authors:** Yunpeng Wang, Jijing Zhao, Kaiyu Han

**Affiliations:** a Department of Respiratory Medicine, The First People’s Hospital of Jiande, Hangzhou, China; b Department of Respiratory Medicine, The Second Affiliated Hospital of Harbin Medical University, Harbin, China.

**Keywords:** histone lactylation, lactic acid, Mendelian randomization, non-small cell lung cancer

## Abstract

The aim of this study was to investigate the effect of lactic acid down-regulation of hexokinase 1 (HK1) on non-small cell lung cancer (NSCLC) through histone lactylation by Mendelian randomization (MR). Genes related to HK1 and NSCLC targets were analyzed using genome-wide association study summary statistics downloaded from public databases. Single-nucleotide polymorphisms associated with HK1 were selected as instrumental variables, and MR analyses were performed using inverse-variance weighted as the primary study method, and MR-Egger, weighted median, simple mode, and weighted mode as complementary methods in order to assess whether HK1 is a protective factor for NSCLC, and to provide further evidence that lactic acid is a risk factor for NSCLC. The results showed that HK1 was significantly and negatively associated with the risk of NSCLC using an inverse-variance weighted method (odds ratio = 0.93, 95% confidence interval = 0.89–0.98, *P* < .05). This suggests that for every 1-unit increase in genetically predicted HK1 expression, the odds of developing NSCLC decrease by approximately 7%. No significant heterogeneity or directional pleiotropy was detected (MR-Egger intercept *P* > .05). Leave-one-out sensitivity analysis confirmed the robustness of the findings. This MR study provides genetic evidence that lactic acid may down-regulate HK1 expression via histone lactylation, thereby increasing the risk of NSCLC. However, limitations include the restriction to European ancestry, limited number of instrumental single-nucleotide polymorphisms due to stringent thresholds, and potential residual pleiotropy not captured by current methods. These factors may affect the generalizability and causal interpretability of the findings.

## 1. Introduction

Non-small cell lung cancer (NSCLC) is the most common type of lung cancer worldwide, accounting for more than 85% of all lung cancer types.^[1]^ Despite significant advances in diagnosis and treatment in recent years, the prognosis of NSCLC remains poor, with a 5-year survival rate of only about 18%.^[2]^ Therefore, it is of great clinical significance to gain an in-depth understanding of the molecular mechanisms of NSCLC and to search for new therapeutic targets.

Lactic acid is one of the end products of cellular metabolism and plays a key role in the tumor microenvironment (TME). Many studies have shown that lactate is not only a source of energy for tumor cells but also promotes tumor growth and metastasis through various mechanisms. For example, lactate can promote angiogenesis by activating hypoxia-inducible factor-1α, which increases the oxygen and nutrient supply to tumors, thus promoting their rapid growth.^[3,4]^ However, the specific mechanism of lactic acid’s action in NSCLC has not been fully elucidated. In recent years, more and more studies have begun to focus on the role of lactate in regulating gene expression in tumor cells. For example, it has been shown that lactic acid can affect gene expression through epigenetic modifications, such as histone lactylation.^[5]^ These findings provide new insights into the possibility that lactic acid may contribute to NSCLC progression by directly regulating the expression of key genes through epigenetic mechanisms.

Hexokinase 1 (HK1) is a key enzyme in the glycolytic pathway and is responsible for catalyzing the conversion of glucose to glucose 6-phosphate, which initiates the glycolytic process. It has been shown that down-regulation of HK1 is associated with malignant progression in a variety of tumors.^[6–9]^ In NSCLC, the expression level of HK1 is closely related to the metabolic status of tumor cells. First, the function of HK1 in NSCLC is mainly reflected in its regulation of the glycolytic pathway. Glycolysis is the main source of energy for tumor cells, and tumor cells tend to produce energy through the glycolytic pathway even under sufficient oxygenation.HK1, as the rate-limiting enzyme of glycolysis, and its expression level directly affects the rate of glycolysis and energy production. Studies have shown that down-regulation of HK1 leads to a decrease in the rate of glycolysis, which in turn affects the energy supply of tumor cells, but the exact mechanism of this effect needs to be further investigated. Secondly, the expression level of HK1 is also associated with the malignant progression of NSCLC. Some studies have shown that down-regulation of HK1 is associated with enhanced invasiveness and metastatic ability of NSCLC. For example, one study found that NSCLC cells with low HK1 expression had higher migration and invasive ability and reduced sensitivity to chemotherapeutic agents.^[10]^ This suggests that down-regulation of HK1 may promote malignant progression of NSCLC by affecting the metabolic state and biological behavior of tumor cells. In terms of regulatory mechanisms, HK1 expression is affected by a variety of factors, including transcriptional regulation, posttranslational modifications (PTMs) and epigenetic modifications. For example, HK1 expression can be regulated by transcription factors such as hypoxia-inducible factor-1α and myelocytomatosis oncogene, which are usually expressed up-regulated in NSCLC and promote HK1 expression.^[11]^ In addition, HK1 expression can be regulated by epigenetic modifications such as histone lactylation. It has been shown that histone lactylation can affect the transcriptional activity of HK1 and thus regulate its expression level.^[12]^

Mendelian randomization (MR) is a method of epidemiological research that uses single-nucleotide polymorphisms (SNPs) as instrumental variables (IVs) to infer causal relationships between exposure factors and outcome variables. Epidemiological research methods of causality can effectively avoid confounders and reverse causality bias because genetic variants are randomly assigned at birth of an individual and are not affected by environmental factors and reverse causality.^[13,14]^

In summary, the functions and regulatory mechanisms of HK1 in NSCLC are complex and diverse, involving multiple aspects of glycolytic pathways, malignant progression and epigenetic modifications. Therefore, in this study, we used MR methods to verify the causal relationship between HK1 and NSCLC expression, and further analyzed the effect of lactate on NSCLC progression. By thoroughly investigating the specific role of HK1 in these processes, it is important to understand the molecular mechanisms of NSCLC and find new therapeutic targets.

This study is the first to evaluate the causal association between HK1 expression and NSCLC using an MR framework system, providing genetic evidence for understanding the pathogenic mechanism of the lactate histone lactylation HK1 axis.

## 2. Methods

### 2.1. Study design

The data HK1 and NSCLC for this study were obtained from the GWAS database (https://gwas.mrcieu.ac.uk/) and the FinnGen cohort (https://storage.googleapis.com/finngen-public-data-r9/summary_stats/finngen_R9_C3_LUNG_NONSMALL_EXALLC.gz). We selected samples from HK1 and NSCLC patients for MR analysis.

The HK1 protein level used in this study was GWAS (GWAS ID: prot-a-1343), which included 3301 European individuals and 10,534,735 SNPs. The FinnGen R9 NSCLC data used in this study included 4901 Europeans and 287,137 controls, with 18,707,596 SNPs.

**Figure 1.**
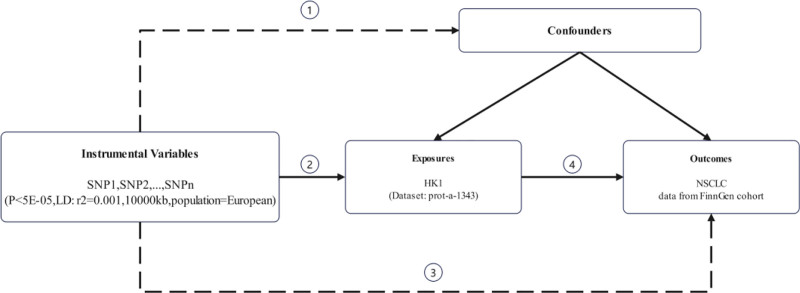
Schematic diagram of the 3 core assumptions underlying the Mendelian-randomization design. ① Independence: the genetic variants (SNPs) are independent of confounders that affect both HK1 expression and NSCLC risk. ② Relevance: the SNPs are robustly associated with HK1 expression. ③ Exclusivity: the SNPs influence NSCLC risk solely through their effect on HK1 and not via alternative biological pathways. ④ MR analyses were performed to test the causal relationship between HK1 and NSCLC. HK1 = hexokinase 1, NSCLC = non-small cell lung cancer, SNP = single-nucleotide polymorphism.

This MR study was based on 3 principal assumptions (Fig. 1). Assumption 1 (Relevance), SNPs affect the risk of NSCLC development only through HK1 expression and not through other pathways. Assumption 2 (Independence), SNPs are not associated with the confounding factors of HK1 and NSCLC. Assumption 3 (Exclusivity), SNPs have an effect on NSCLC by regulating HK1, and have no direct correlation with the NSCLC incidence risk were not directly associated.

### 2.2. MR analysis

Forest maps were drawn using the grid package (version 4.4.1) and the forestploter package (version 1.1.2) in the R software (version 4.4.1, R Foundation, Vienna, Austria). MR analyses were performed using the TwoSampleMR package (version 0.6.8, MRC Integrative Epidemiology Unit , University of Bristol, Bristol, UK).

In the MR analysis, we used inverse-variance weighted as the primary study method to assess the potential causal effect of HK1 on NSCLC. Because of the small sample size of HK1, SNPs with *P* < 5e−05 were selected as IVs for the exposure factors and *P* < 5e−08 for the outcome variables. To minimize possible confounders caused by genetic background variation, we selected individuals of European ancestry, which improved the robustness and reliability of the study results. Figure 1 illustrates the study design and the underlying assumptions of MR used in the study. MR-Egger was used to detect horizontal pleiotropy. To assess horizontal multidirectional effects, we used the MR-PRESSO test to identify outliers and assess potential multidirectional effects of genetic variation in IVs. If outliers were detected, causality was reassessed. The *F*-statistic was calculated to assess the strength of the instrument and to observe whether the *F*-value exceeded 10 to minimize the bias caused by weak instruments.^[15]^ In addition, we used funnel plots to check for bias and heterogeneity in the results and assessed model performance using the leave-one-out method. *P*-values <.05 were considered statistically significant, but causal inference requires further validation.

## 3. Results

**Figure 2.**
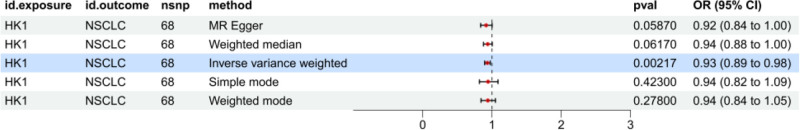
Forest plot showing the causal effect of genetically predicted HK1 expression on the risk of developing NSCLC. Results are presented for IVW, MR-Egger, weighted median, simple mode, and weighted mode estimators. The overall IVW odds ratio was 0.93 (95% CI = 0.89–0.98, *P* < .05), indicating a significant protective effect of higher HK1 expression against NSCLC. CI = confidence interval, HK1 = hexokinase 1, IVW = inverse-variance weighted, MR = Mendelian randomization, NSCLC = non-small cell lung cancer, OR = odds ratio.

**Figure 3.**
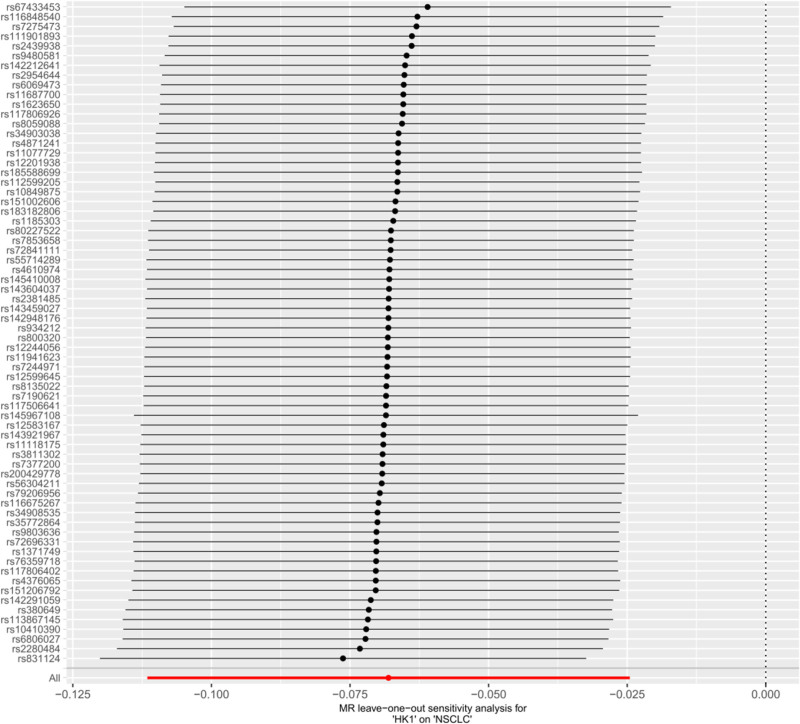
Leave-one-out sensitivity analysis. Each point represents the IVW estimate when a single SNP is removed from the analysis. The vertical dashed line indicates the overall IVW estimate using all SNPs; the absence of large deviations demonstrates that no single genetic variant disproportionately influences the causal estimate. HK1 = hexokinase 1, IVW = inverse-variance weighted, MR = Mendelian randomization, NSCLC = non-small cell lung cancer, SNP = single-nucleotide polymorphism.

**Figure 4.**
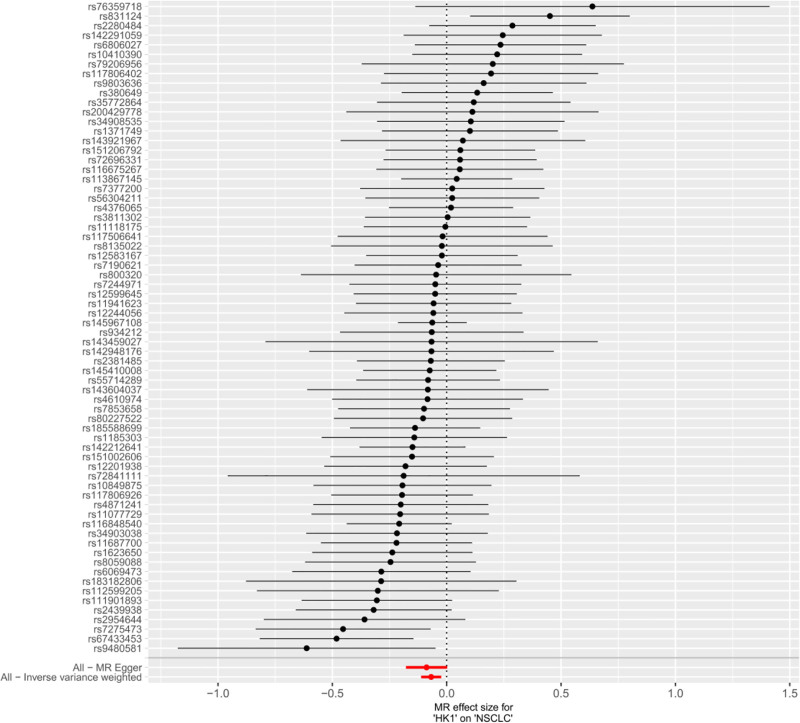
Forest plot of the individual SNP-level causal estimates for the effect of genetically predicted HK1 expression on NSCLC risk. Each horizontal line depicts the per-allele log odds ratio (log OR) of developing NSCLC conferred by 1 instrumental SNP. Visual inspection reveals that the majority of individual SNP estimates lie to the left of the null line, supporting a consistent protective effect of higher HK1 expression against NSCLC across instrumental variables. HK1 = hexokinase 1, MR = Mendelian randomization, NSCLC = non-small cell lung cancer, SNP = single-nucleotide polymorphism.

**Figure 5.**
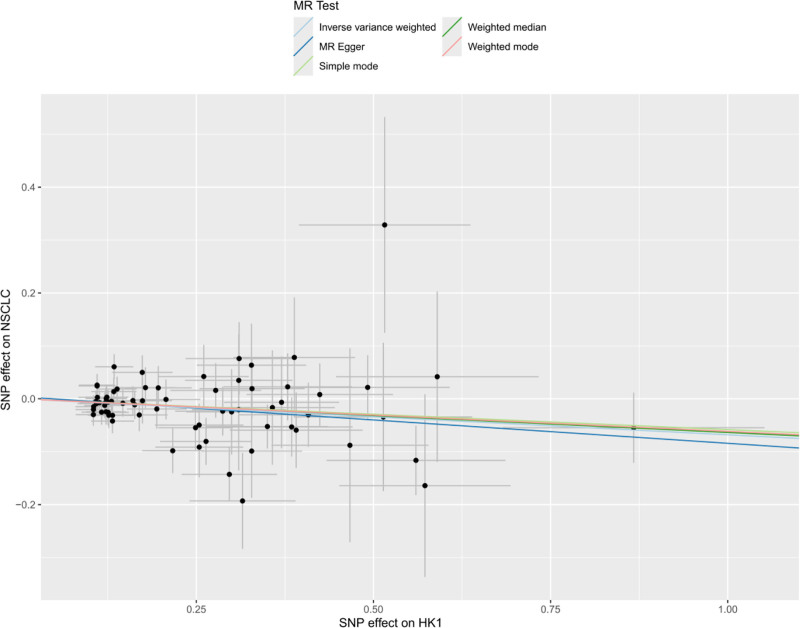
Scatter plot of SNP-HK1 associations (*x*-axis) against SNP-NSCLC associations (*y*-axis). Each point corresponds to a single SNP; error bars indicate 95 % confidence intervals. The slope of the fitted IVW line represents the causal effect of HK1 on NSCLC risk. HK1 = hexokinase 1, IVW = inverse-variance weighted, MR = Mendelian randomization, NSCLC = non-small cell lung cancer, SNP = single-nucleotide polymorphism.

**Figure 6.**
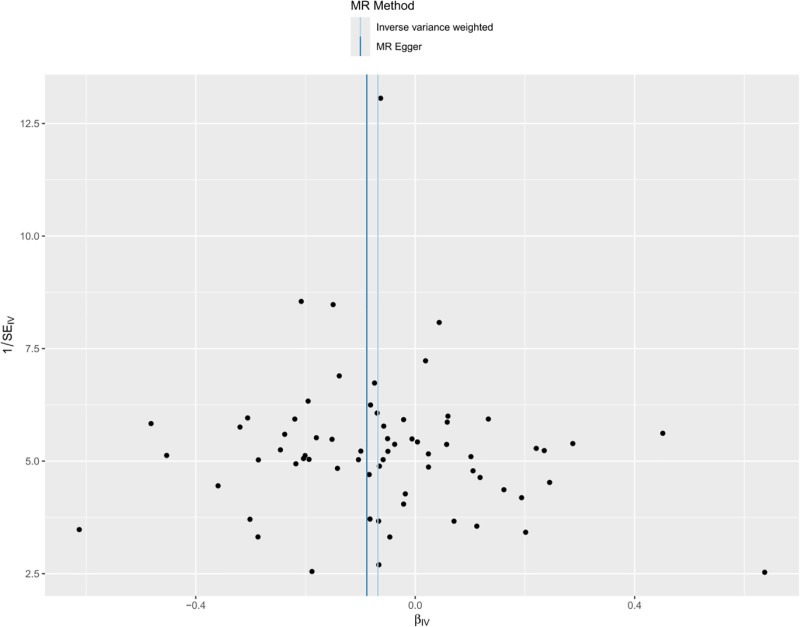
Funnel plot assessing directional pleiotropy and heterogeneity. Plotted are the SNP-specific causal estimates against their precision (1/standard error). Symmetrical distribution around the IVW point estimate suggests minimal directional pleiotropy and no substantial small-study bias. IVW = inverse-variance weighted, MR = Mendelian randomization, SNP = single-nucleotide polymorphism.

It has been shown that lactic acid can down-regulate HK1 expression through histone lactylation, and the down-regulation of HK1 is associated with malignant progression of NSCLC.^[16]^ Therefore, in this study, we used MR method to verify the causal relationship between HK1 and NSCLC expression and further analyze the effect of lactic acid on NSCLC progression. Inverse-variance weighted method suggested a significant negative correlation between HK1 and the risk of developing NSCLC (odds ratio = 0.93, 95% confidence interval = 0.89–0.98, *P* < .05), and The other 4 methods yielded different conclusions (*P* > .05) (Fig. 2). In addition, no multidirectional SNPs were found to require correction by the MR-PRESSO outlier test. Throughout the MR study, *F*-values were >10, indicating good instrumental strength. The model was cross-validated using the leave-one-out method, and no single SNPs had a large impact on the overall results (Fig. 3). A forest plot of the effect of each SNPs on the results is shown in Figure 4. Scatter plots (Fig. 5) and funnel plots (Fig. 6) of the MR analyses showed that HK1 was causally associated with a reduced risk of developing NSCLC.

## 4. Discussion

PTMs of proteins can alter the biochemical properties of precursor proteins by adding chemical groups to one or more amino acid residues to give them specific functions. Common modifications of PTMs include phosphorylation, acetylation, ubiquitination, methylation, glycosylation, etc.^[17]^ In 2019, Zhang et al^[18]^ reported for the first time a new type of lactate-induced PTM-lactylation, and they found that lysine residues in histone tails could undergo lactylation. Subsequent studies confirmed that lactylation is prevalent in a wide range of cancers and is closely associated with processes such as the development of malignant tumors. It has been shown that lactic acid can affect a variety of proteins, including SOX9,^[19]^ IGF1R,^[20]^ LKB1,^[21]^ and H3K18,^[22]^ through histone lactylation, which plays a key role in the proliferation and metabolism of lung cancer cells.

Lactate concentration in normal human serum is 1.5 to 3 mmol/L, whereas in tumor patients, it can rise to 10 to 30 mmol/L, and can even reach 50 mmol/L within the tumor.^[23]^ Studies^[24,25]^ have demonstrated that high concentrations of lactate in the TME are associated with poor clinical prognosis, such as lymph node or distal metastasis and low survival in a variety of tumors. Lactic acid in the TME can promote tumor cell proliferation, angiogenesis, metastasis, drug resistance, and immunosuppression through a variety of mechanisms.

Jiang et al^[16]^ found that when NSCLC cells were treated with lactic acid, the level of histone lactylation was increased and the transcription of HK1 was down-regulated, suggesting that lactic acid increased histone lactylation of the HK-1 promoter. We therefore used MR methods to verify the causal relationship between HK1 and NSCLC expression. MR provided a genetic basis for the results, which showed a significant negative correlation between HK1 and the risk of developing NSCLC, suggesting that lactic acid downregulates HK1 through histone lactylation, which can promote NSCLC progression.

In this study, we employed the MR method, which is inherently less prone to confounding factors and biases, thereby strengthening the causal inference. Our analyses were confined to individuals of European ancestry, minimizing the risk of population structure bias and ensuring the robustness of our findings.

However, it is important to acknowledge the limitations of the MR analyses conducted in this study. First, although we excluded potential pleiotropic SNPs and MR-Egger regression indicated minimal horizontal pleiotropy, pleiotropy bias remains a concern. Second, while the MR-PRESSO analysis did not detect any outliers, the possibility of undetected outliers cannot be entirely ruled out. Third, owing to the limited number of SNPs meeting the stringent inclusion criteria for HK1 (*P* value < 5e−05, *R*^2^ = 0.001 with kb = 10,000), we had to slightly relax these criteria, which may introduce a degree of false positives. Fourth, the analyses presented here are based on individuals of European ancestry, and further research is needed to determine whether these findings are generalizable to other populations. Fifth, we are unable to correct for the same confounders for both MR and observational studies since MR uses integrated data and observational studies use individual data. Sixth, selection bias may have been introduced because both the exposure GWAS (HK1 protein levels) and the outcome GWAS (NSCLC) were restricted to individuals of European ancestry who volunteered for biobank-based cohorts. Such participants tend to be healthier and of higher socioeconomic status than the general population, potentially leading to collider bias and limiting the external validity of our findings.

## 5. Conclusion

This MR study provides preliminary genetic evidence that higher genetically predicted HK1 expression may be associated with a modestly lower risk of NSCLC among individuals of European ancestry. The data are compatible with, but do not directly demonstrate, the hypothesis that lactic acid influences NSCLC risk through HK1-related pathways. Owing to the strict European-ancestry sampling, the limited number of instrumental SNPs, and the absence of direct measurements of histone lactylation or lactate levels, these findings cannot be generalized to other populations nor interpreted as proof that lactic acid downregulates HK1. Mechanistic studies in diverse ancestries are required before any clinical inferences can be drawn.

This manuscript meets the STROBE-MR guidelines for Mendelian-randomization reporting.

## Acknowledgments

The genetic association estimates for HK1 and NSCLC were derived from a comprehensive genome-wide association meta-analysis conducted by the FinnGen cohort. The authors extend their gratitude to all contributing investigators for their invaluable data contributions.

## Author contributions

**Conceptualization:** Yunpeng Wang.

**Data curation:** Yunpeng Wang, Jijing Zhao.

**Formal analysis:** Yunpeng Wang.

**Funding acquisition:** Yunpeng Wang, Jijing Zhao.

**Investigation:** Yunpeng Wang.

**Methodology:** Yunpeng Wang.

**Project administration:** Yunpeng Wang.

**Resources:** Yunpeng Wang, Kaiyu Han.

**Software:** Yunpeng Wang.

**Supervision:** Yunpeng Wang, Kaiyu Han.

**Validation:** Yunpeng Wang, Kaiyu Han.

**Visualization:** Yunpeng Wang.

**Writing – original draft:** Yunpeng Wang.

**Writing – review & editing:** Yunpeng Wang.
